# Transdiagnostic skills training group of dialectical behavior therapy: a long-term naturalistic study

**DOI:** 10.1186/s40479-023-00243-y

**Published:** 2023-12-21

**Authors:** Amaury Durpoix, Enzo Lachaux, Luisa Weiner, Sébastien Weibel

**Affiliations:** 1 Psychiatry, Mental Health and Addictology Department, University Hospitals of Strasbourg, 1 place de l’Hôpital, Strasbourg, 67000 France; 2 Inserm u1114, Strasbourg, 67000 France; 3 Faculty of Medicine, Strasbourg University, Strasbourg, 67000 France; 4 Faculty of Psychology, Strasbourg University, Strasbourg, 67000 France; 5 Laboratoire de Psychologie des Cognitions, Strasbourg University, Strasbourg, 67000 France

**Keywords:** Dialectical behavior therapy, Emotion regulation, Skills training, Transdiagnostic, Borderline personality disorder, Bipolar disorder, Attention deficit/hyperactivity disorder, Emotion dysregulation

## Abstract

**Introduction:**

Dialectical Behavior Therapy (DBT) has assembled a large body of evidence for the treatment of emotional dysregulation in borderline personality disorder (BPD), but also in other disorders characterized by emotional dysregulation (e.g., bipolar disorder (BD) and ADHD). Standalone skills learning groups address the problem of limited resources in several clinical settings. Furthermore, transdiagnostic skills groups facilitate the recruitment and decrease the scattering of resources in psychiatric settings. However, few studies have focused on the pertinence of transdiagnostic standalone skills groups in naturalistic settings as well as their long-term outcomes. The goal of this study is to assess the impact of participation in a transdiagnostic DBT skills group one year after its completion.

**Method:**

Transdiagnostic DBT skills training groups were provided for BPD, BD and ADHD patients in a University Psychiatric Department (Strasbourg, France), between 2019 and 2020. They consisted of 16 group sessions of 2.5 h and 3 individual sessions. At 1-year follow-up, ad-hoc questionnaires were proposed to all participants to assess the perceived impacts, the changes in symptomatology, and the maintenance of skills learned.

**Result:**

22 of the 31 participants were interviewed at the one-year post-group session (64% BPD, 41% ADHD and 27% BD). 73% participants estimated that group impact was important or very important, 64% stated using the skills learned often or very often, mainly emotion regulation skills. An improvement in emotional instability, substance use, impulsivity and suicidal thoughts was reported by respectively 100%, 91%, 86% and 85% of participants. Quality of life improved according to 90% participants. All patients reported an improvement in suicidality during the post-group year, especially in suicide attempts. Psychotropic medication decreased in 59% of participants.

**Discussion:**

Our one-year naturalistic study suggests that transdiagnostic DBT skills training groups are promising for the treatment of emotional dysregulation in people with BPD, BD and/or ADHD. The observational design and the lack of control group are the main limitations. Randomized controlled studies are required to confirm the long-term efficacy of transdiagnostic skills learning groups in naturalistic settings.

## Introduction

Emotion dysregulation (ED) is defined as an inability to use effective strategies to reduce intense emotional oscillations. ED is a core processus of borderline personality disorder (BPD) but it is also observed in other disorders such as bipolar disorder (BD) [1], ADHD [2], or autism [3]. Importantly, ED is associated with suicidal behaviors, poorer social and interpersonal functioning, and psychological distress [4, 5].

Amongst psychosocial interventions for BPD, Dialectical Behavior Therapy (DBT) has the highest evidence level of efficacy [6]. DBT targets ED [7, 8], notably by teaching emotion regulation skills. Linehan’s biosocial theory, the theory underlying DBT, posits ED as the core mechanism of BPD symptoms. According to Linehan’s theory, ED arises from interactions between emotional vulnerability and an invalidating developmental environment [9]. Mechanisms that explain emotional dysregulation may differ to some extent across disorders and ED has been reported to be heightened in BPD compared to BD [1] and ADHD [2]. However, several recent findings suggest that ED is a transdiagnostic mechanism of psychopathology [10–12] and, beyond its clinical relevance, Linehan’s biosocial model has gathered empirical evidence supporting its pertinence in BPD and in other psychiatric and neurodevelopmental disorders [3, 13].

Importantly, several studies have shown that DBT is also effective in targeting ED found in disorders other than BPD [14–16], such as BD [17–19], and ADHD [20]. No other psychotherapy has demonstrated similar levels of evidence for treating ED. Furthermore, even though pharmacological treatments are recommended as first-line approaches in BD and ADHD, their effectiveness for ED is small to moderate [11].

Comprehensive DBT is an intensive therapy that includes four components (skills training group, individual therapy, phone coaching and team consultation), needing sufficient staff and patient availability, which limits its implementation [21, 22]. On average, 45.3% of teams that have implemented DBT fail to pursue the delivery of the therapy, usually after 2 to 5 years [22]. These interruptions are dramatic given that the number of DBT therapists is low compared to patients’ needs [23]. For this reason, less resource-intensive and more easily implementable models have been evaluated, in particular those focusing on standalone DBT skills training groups [24]. DBT skills training groups is the most investigated standalone component of adapted DBT models. Recent meta-analyses found 4 randomized controlled studies for BPD with standardized mean difference of -1,05 on affective instability [25] and 12 for other disorders with large overall effect size [26].

DBT has also been adapted for patients with ED, regardless of their main diagnoses, allowing to have only one group for different diagnoses instead of several groups for each diagnosis. To our knowledge, 4 studies exist on transdiagnostic DBT groups. The only randomized controlled study [27] found that after a 4-month DBT group, patients with depression and/or anxiety disorder experienced a decrease in ED and anxiety with an important effect size compared to the control group. A clinical improvement was also found by other studies in depression and anxiety after a 5-week DBT group with daily sessions [28]. Improvements have also been reported in deliberate self-harm after a 6-week DBT group with daily sessions [29], and in emotion regulation after a 6-month DBT group with weekly sessions [30]. However, these studies focused mainly on depression and anxiety disorder. Transdiagnostic groups have been less studied in BD, BPD and ADHD. Yet, the latter are often comorbid and characterized by high levels of impulsivity and ED, which are linked to suicidal behaviors [1, 15, 31]. Moreover, studies on transdiagnostic DBT groups did not analyze outcome follow-up data beyond a 3-month period.

In this study, we were interested in the long-term sustainability of treatment benefits of skills learning DBT transdiagnostic groups targeting ED. While there is no consensus on the definition of a long-term effect in psychotherapeutic treatment, we considered assessing the benefits one year after treatment completion as an adequate reflection of the maintenance of effects. Indeed, given that BPD, BD and ADHD are characterized by their lifelong course, the sustainability of the effect is crucial when recommending relatively brief treatments. Furthermore, for it to be clinically relevant, this benefit must impact both the consequences of ED, such as suicidal ideation, and quality of life. We also explored the maintenance of skills use, which could indicate that clinical improvements are related to this assumed active principle of DBT.

Given the methodological challenges of conducting a controlled trial in a naturalistic setting, our study employed a retrospective method, and we queried patients about their perceived improvement. Our primary hypothesis was that one year after treatment, patients would report improvements in symptoms associated with ED and in their quality of life, on the one hand, and that they would also report a continued use of the DBT skills learned during the groups, on the other. We hypothesized that the observed improvement would be associated with the generalization and continued use of skills.

## Method

### Ethical aspects

Participants were individually informed through written document that their data could be used anonymously to evaluate the program and that they were assured that they could decline to participate without incurring any negative consequences. This research received approval by the Ethics Committee of the Strasbourg Medicine Faculty (CE-2021-108).

### Characteristics of participants & setting

Transdiagnostic DBT skills training groups were provided to patients with BPD, BD or ADHD in the Psychiatry Department of Strasbourg University Hospital. Patients were referred by psychiatrists, the referral criteria being a diagnosis of BPD, BD and ADHD associated with ED. Diagnosis was done according to the DSM-5 criteria for each disorder.

One year after the last session of transdiagnostic DBT groups, a follow-up individual session was systematically proposed to patients, during which a semi structured interview was administered, described in the “outcome measures” section (questionnaire available as Supplementary material).

Between March 2018 and December 2019, 4 transdiagnostic DBT groups were conducted with 31 different patients, including 3 who prematurely dropped out. Out of the remaining 28 participants, 6 did not attend the 1-year follow-up debriefing session between 2019 and 2020 (4 renewed their participation in DBT group before this debriefing and were therefore not included, and 2 did not reply). Therefore, 22 patients responded to the 1-year questionnaire. In the referral letters, the main diagnosis was BPD (64%), ADHD (41%) and BD (27%). At least 2 diagnoses were mentioned for 5 patients (23%) (Table 1).

**Table 1:** Characteristics of participants and their care

	All diagnosis (N = 22)	BPD (N = 14)	ADHD (N = 9)	BD (N = 6)
**Socio-demography**				
**Age**	Years: 33.3	30.2	37	34.7
**Sex**	Women: 91%	100%	89%	83%
**History**	Self-harm: 55%	71%	22%	50%
	Suicide attempts: 50%	57%	33%	67%
	Psychiatric hospital: 55%	57%	44%	67%
**Comorbidity** BPD/ADHD/BD in referral letter	3 diagnoses: 9%	14%	22%	33%
	2 diagnoses only: 14%	14%	22%	33%
		BPD + BD 14% BPD + ADHD 14% ADHD + BD 14%		
**Care at 1-year post-DBT**				
**Psychotherapist**	Psychiatrist: 91%	93%	89%	100%
	Psychologist: 50%	50%	11%	50%
Orientation	DBT: 27%	36%	12%	33%
	CBT: 27%	14%	44%	33%
	Other: 46%	50%	44%	33%
Medication	No: 23%	21%	11%	17%
	Lithium: 23%	29%	22%	50%
	Lamotrigine: 41%	50%	44%	67%
	Atypic antipsychotic: 36%	36%	56%	67%
	Antidepressant: 23%	29%	11%	0%
	Methylphenidate: 27%	14%	56%	33%
	Anxiolytic/sedative: 18%	29%	11%	17%

### Intervention: transdiagnostic DBT groups of 16 weeks

Based on Neasciu & al [27, 32], the transdiagnostic DBT skills training groups consisted of 16 weekly sessions of 2.5 h. Divided in 2 cycles with a fixed skills learning program (Table 2), it covered the four DBT modules developed by Linehan [8] : mindfulness, emotion regulation, distress tolerance and interpersonal effectiveness. Each cycle began with mindfulness teaching sessions, participants could enter the program during these sessions, i.e. sessions 1, 2 and 10. Two debriefing sessions were held at the end of each cycle and another one, one year after treatment completion. These debriefing sessions provided an opportunity to review the skills learned in the previous sessions, engage in role-play or skills modelling, address questions, assist participants in noticing their progress, and gather feedback from participants regarding their experiences in the group sessions. This program was named GREMO – in French : Groupe de Régulation Emotionnelle [33, 34]. In addition to DBT skills training groups, a consultation team was held weekly and patients had treatment as usual (psychiatric consultation, medication, individual psychotherapy…). Therapists were psychiatrists or clinical psychologists trained in DBT (i.e., the four authors).

**Table 2:** List of taught DBT skills

Cycle	Modules	Sessions	Skills
**Cycle 1**	**Mindfulness**	1	Wise mind and the “what” skills of mindfulness
		2	The “How” skills of mindfulness
	**Distress Tolerance**	3	STOP & TIP skills
		4	ACCEPTS skills
		5	IMPROVE and self-soothing skills
		6	Radical acceptance and willingness
	**Interpersonal Effectiveness**	7	DEAR MAN skill
		8	GIVE and FAST skills
		9	Validation others and self-validation
***Debriefing session of cycle 1***			
**Cycle 2**	**Mindfulness**	10	Mindfulness Skills Review
	**Emotional Regulation**	11	Understand, identify and name emotions
		12	Fact-checking
		13	Opposite action
		14	Problem solving
		15	ABC skills
		16	PLEASE skills
***Debriefing session of cycle 2***			
***Debriefing session at one year***			

### Outcome measures, design & aim

An individual debriefing session at 1-year post-group was done through semi-structured interview guided with an ad hoc questionnaire. The semi-structured interview aided the recall of the post-treatment year to improve the reliability of responses. The questionnaire (see supplementary materials) was divided in three parts. The first one concerned DBT skills: impact of skills training group, frequency of skills use, the most used skills (mindfulness, emotional regulation, distress tolerance or interpersonal effectiveness). The second focused on perceived changes for specific symptoms (emotional instability, depression, sleeping, suicidal thoughts, impulsivity, substances use, eating behavior, emptiness feeling, loneliness feeling), overall quality of life and in specific domains (couple life, friendship life, spiritual life, professional life, studies/training, leisure, health, hope, meaning of life, self-esteem). Responses were rated according to a 7-point Likert scale (very highly improved, highly improved, slightly improved, stable, slightly worsened, highly worsened, very highly worsened) or “non-applicable”. The third part focused on suicidality and use of services (psychiatric hospitalization, suicide attempt, self-harming behavior, consultations with clinicians, and psychotropic medication take).

Following the debriefing sessions held between 2019 and 2020, we analyzed retrospectively this questionnaire as part of this naturalistic study. The aim was to assess the one-year impact of a transdiagnostic 4-month DBT skills training group for BPD, BD and/or ADHD.

### Statistical analysis

Descriptive analyses were performed on the responses to the questionnaire, mostly expressed in proportions. To explore the relationship between emotion instability and suicidal ideation on the one hand, as well as the relationship of these symptoms with socio-demographic characteristics, skills use, and psychotherapeutic follow-up the year after the group on the other hand, Fisher exact tests were performed as the variables were categorical. Since these were exploratory analyses conducted on a clinical sample without prior power estimation, no correction for multiple testing was applied, and the results should be interpreted accordingly. These statistical analyses were performed using IBM SPSS Statistics 27 software.

## Results

### Perception of change at the one-year debriefing session

#### Perception of DBT skills training impact at 1 year

**Fig. 1:**
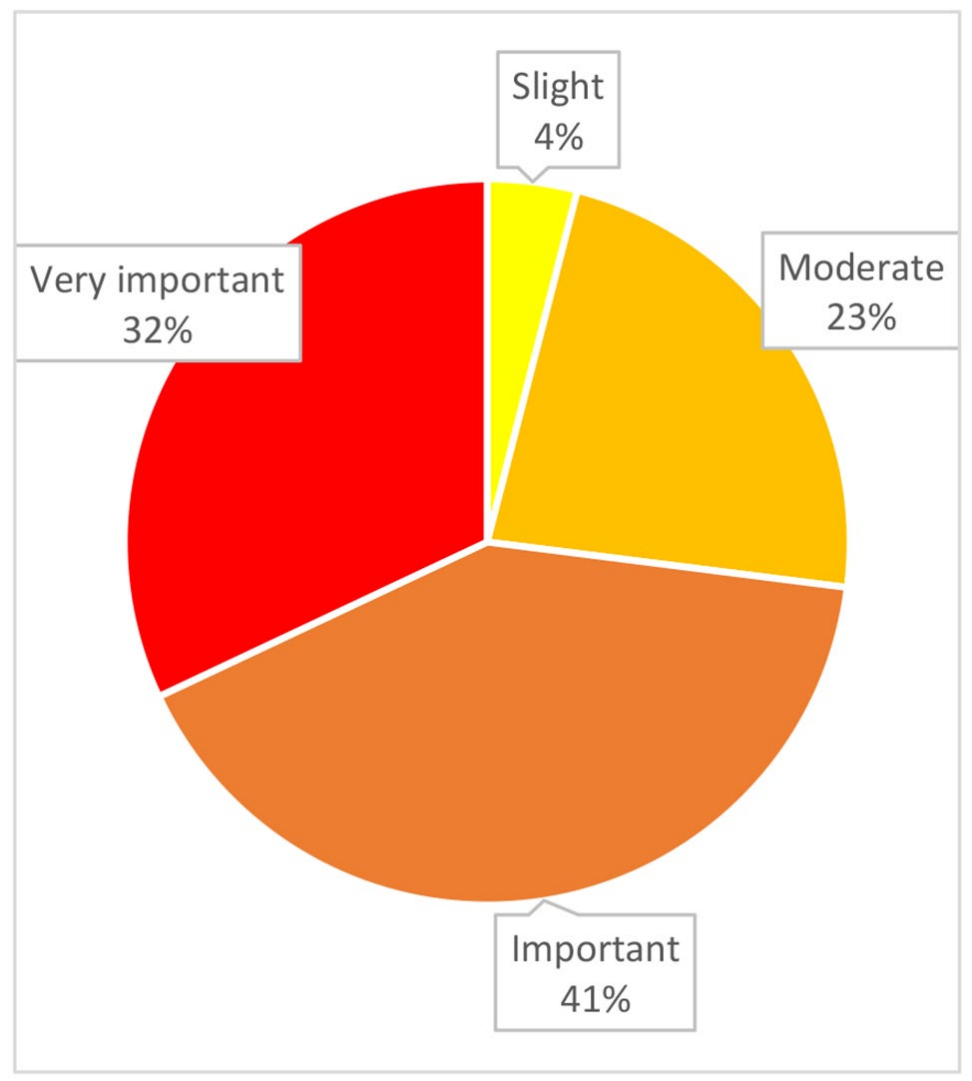
Participants’ subjective estimation of the impact of DBT skills training at 1 year (*N* = 22)

**Fig. 2:**
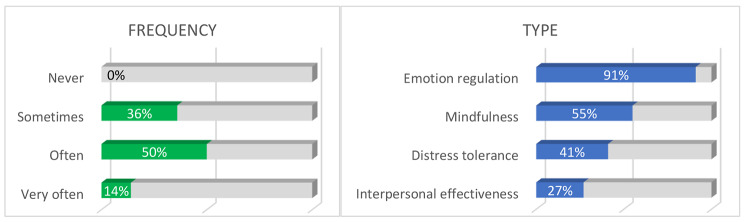
Participants’ assessment of skills uses over the post-group year (*N* = 22)

DBT skills training impact was estimated as important or very important by 73% of participants (Fig. 1). One year following the last session, 64% stated using the skills learned often or very often, emotional regulation skills being the most used according to the participants (Fig. 2).

#### Clinical change at 1 year

**Fig. 3:**
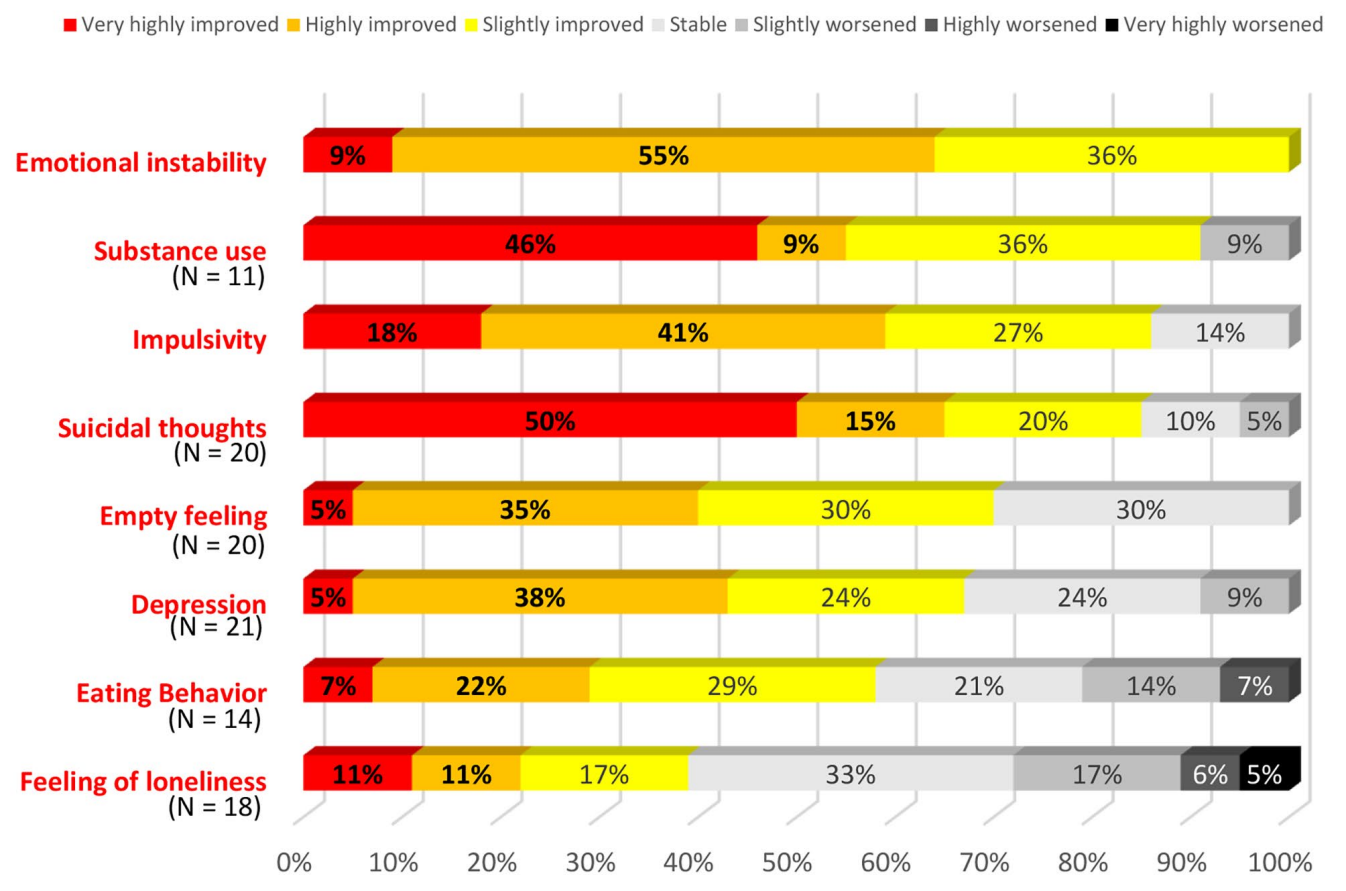
Participants’ assessment of symptom change at 1-year follow-up (*N* = 22)

At 1-year post-intervention, 100% of participants reported experiencing an improvement of emotional instability, 91% of substance use (for the 11 concerned participants), 86% of impulsivity and 85% of suicidal thoughts (for the 20 concerned participants). Improvements in feelings of emptiness, depression, eating behavior and feeling of loneliness were less frequently noticed (Fig. 3).

**Fig. 4:**
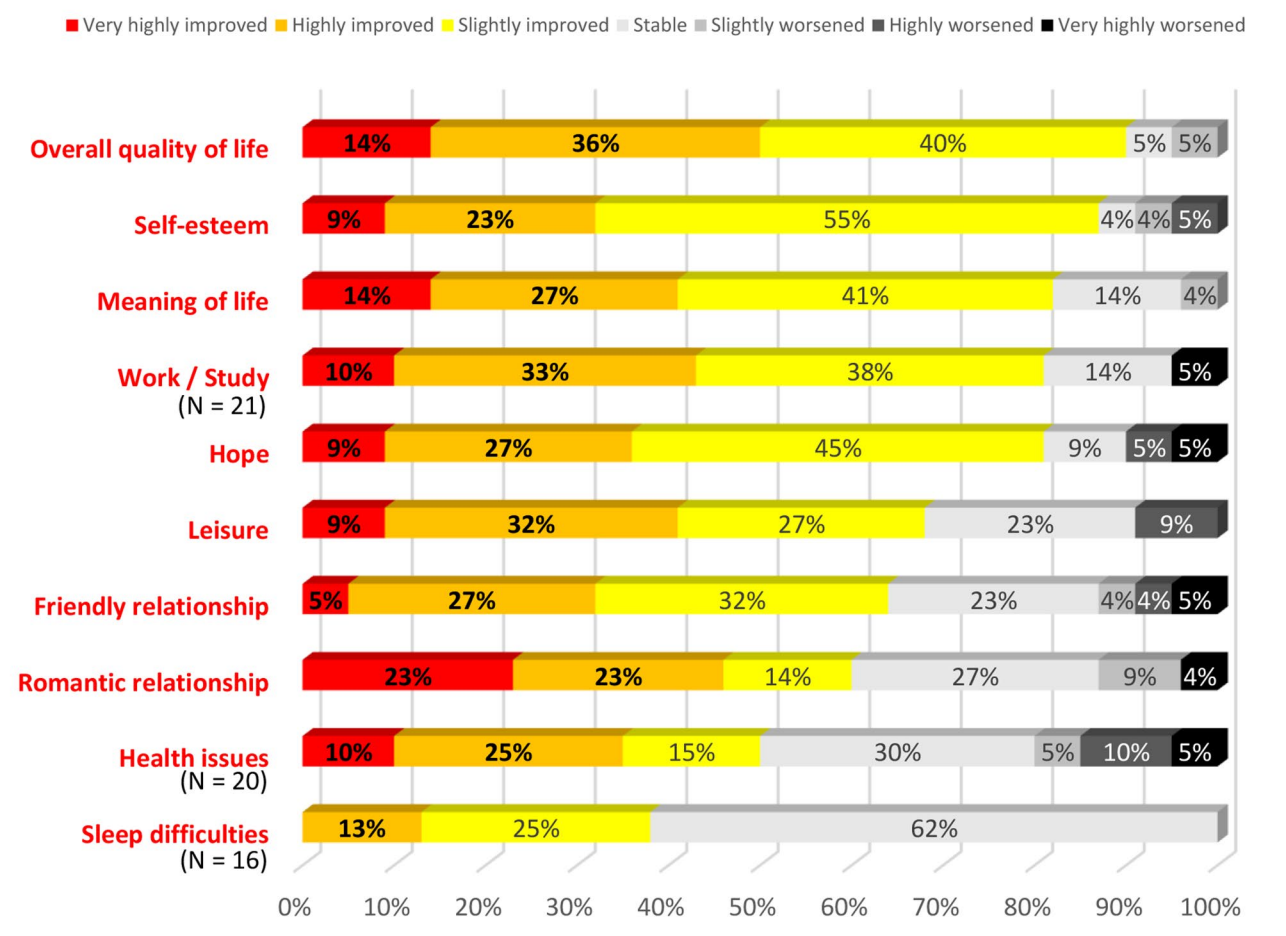
Participants’ assessment of the evolution of their quality of life at 1-year follow-up (*N* = 22)

According to our survey, 90% of participants reported an increase in their overall quality of life. More specifically, 85% experienced improvements in self-esteem, 82% in the meaning given to their life, 81% in work/study and hope, 68% in leisure, 64% in friendly relationship, 60% in romantic relationship, 50% in health issues, and 38% in sleeping difficulties (Fig. 4).

#### Perceived change of suicidality and use of services at one-year post-DBT

**Fig. 5:**
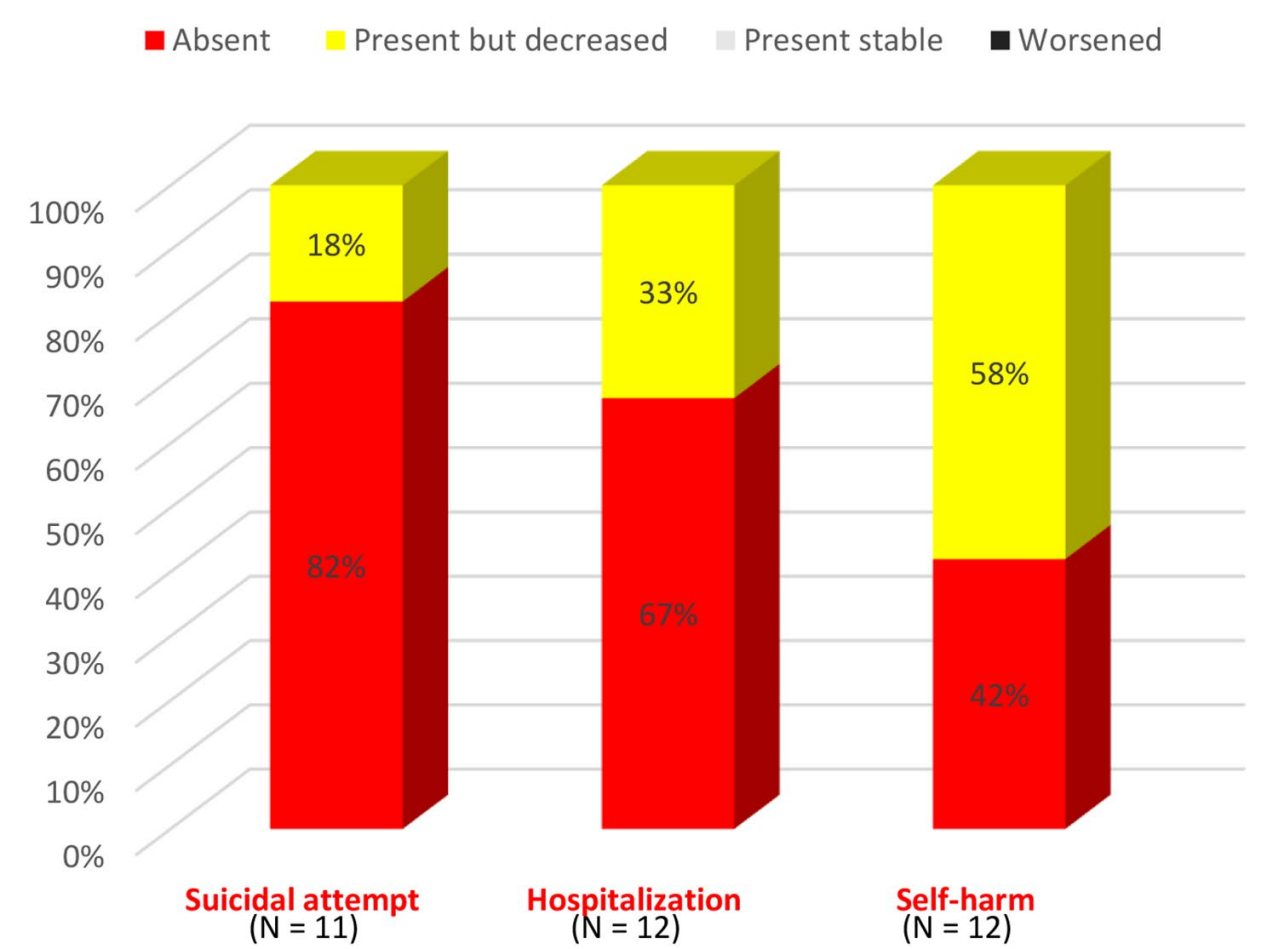
Participants’ assessment of the change of suicidality at 1-year follow-up

All patients reported an improvement in suicidality if they were concerned, especially in suicide attempts (Fig. 5). None reported stabilization or worsening.

Regarding their medication, 13 patients said that it was decreased (59%), 3 patients that it was increased (14%) and 6 that it was stable (27%). Five patients were not taking any medications (23%).

Regarding the consultations with psychotherapist, the frequency decreased according to 50% of patients and remained stable according to others. This frequency was once a year for two patients (9%), one to three times a month for 10 (45,5%), and once a week for 10 (45,5%).

#### Predictors of improvement

According to the exact Fisher test, improvement in emotional instability or suicidal ideation was not associated with socio-demographic characteristics (sex, age or disorder), nor with the type of individual therapy and its intensity during this post-group year. By contrast, the frequency of skills use was associated with improvement in emotional instability (*p* = 0.014). Furthermore, improvement in emotional instability and improvement in suicidal ideation were correlated (*p* = 0.010). Nevertheless, the frequency of skills use was not associated with improvement in suicidal ideation.

## Discussion

This naturalistic study aimed to explore the one-year impact of a transdiagnostic 4-month DBT skills training group for BPD, BD and/or ADHD. To our knowledge, our study is the first to investigate the long-term impact of a transdiagnostic DBT group. One-year post-treatment, all participants deemed that the DBT skills training group had had a positive impact, which was rated as important or very important by three quarters of them. All participants reported improvements in emotional instability, and over 85% in substance use, impulsivity, and suicidal thoughts. Although other symptoms showed less improvements, more than half of participants noticed improvements in eating behavior, depression, feelings of emptiness and over a third in feelings of loneliness. For each assessed symptom, participants reported more improvements than stability or worsening. Improvements were also reported by most patients for quality of life, suicidal behaviors, and use of services.

Consistent with other studies [35, 36], the use of DBT skills was associated with improvement in emotional instability. Moreover, this improvement was correlated with suicidal thoughts improvement. In contrast, none of these symptoms were correlated with patients’ socio-demographics characteristics nor with individual therapy attended during the post-group year. Half of participants reported a decrease in the frequency of sessions with psychotherapists and the other half remained stable. Participants reported using mainly emotion regulation skills. This is in contrast with findings suggesting that during the course of the group, the most used skills are usually distress tolerance ones [37]. The absence of correlation between socio-demographics characteristics and emotional instability improvement suggests that regardless of age, sex or diagnosis, emotional stability improves after the transdiagnostic DBT group.

The literature on DBT stand-alone transdiagnostic groups is recent and scarcely developed. The Neasciu et al. study [27], which inspired our DBT program, included anxious or depressed patients and excluded those with high suicidality. Like Neasciu et al., we observed an improvement in ED associated with the use of DBT skills. However, the authors found it at post-therapy and at two months follow-up. Like Gibson et al. [29], who evaluated a 6-week daily group program in a sample with 18,6% BD and 74,3% BPD individuals, we found an improvement in self-harming behaviors, although their post-group follow-up was held at three months only. Other short transdiagnostic DBT groups found clinical improvements in emotion regulation, depression and hope, but a follow-up was not provided [28, 30]. Therefore, our study adds to this literature by providing evidence for longer-term effects (up to a year) of a short DBT skills training group in people with BD, BPD and or/ADHD.

The acquisition of DBT skills is readily adaptable to various diagnostic contexts, particularly considering that Linehan’s biosocial model can account for the development of ED in the case of developmental peculiarities [3]. Furthermore, DBT skills are now widely disseminated and have become a dominant model. However, our data do not allow us to determine whether DBT skills in particular are effective or if considering ED in various disorders and assisting patients in regulating their emotions, regardless of the therapeutic model, could yield similar results. Taking these factors into account, it is certainly valuable to use the DBT model in standalone group formats, although other modalities deserve to be tested and compared.

Our results suggest that it is likely beneficial to offer DBT skills-training groups to patients with various psychiatric disorders, and this treatment modality can be provided using a transdiagnostic approach. Based on our clinical experience, the presence of patients with different diagnoses has not been a hindrance for the participants, but rather an opportunity for interaction and learning. This observation seems corroborate by the results of the study, notably few dropped-out. Qualitative studies would provide a deeper exploration of this aspect and the overall experiences of the participants. In addition to being consistent with Linehan’s [7] behavioral approach of ED, transdiagnostic formats make it easier to recruit an adequate number of patients without focusing on the primary diagnosis, particularly for non-specialized psychiatric setting. Furthermore, the cost of such programs is relatively modest, within the scope of traditional outpatient psychiatric care services, as it can be implemented without significant organizational modifications. Studies evaluating the cost-benefit ratio should be conducted with larger samples.

Several significant limitations of our study should be noted. First, the absence of a control group does not allow us to conclude that the DBT skills training group is the active ingredient of clinical improvement. While emotional dysregulation in ADHD and BD is usually considered stable over 1–2 years without therapeutic intervention, this is less consensual in BPD because remission in BPD symptoms reaches 39% at 2 years [38–40]. However, this did not mean complete regression of all symptoms or absence of relapse risk [41]. At one-year, the control groups referenced in Finch’s meta-analysis [42] had low effect size on BPD symptoms and nearly zero on suicidality. Second, the retrospective self-reported data may have been influenced by a social desirability bias and a memory bias. These biases may have overestimated the results. However, the evolution is not the same for all symptoms, which suggests that these biases did not explain all the improvements found here. Third, the small sample limits the reliability of Fisher exact test to determine if some characteristics of the participants could have moderated the response of the intervention (age, initial severity of ED, diagnosis). The sample consists of 86% women, and we were unable to draw conclusions about a gender-related treatment effect. Fourth, individual DBT sessions was offered to some of the patients presenting with severe BPD symptoms. Thus, we cannot affirm that the skills group stand-alone is sufficient for all patients presenting with severe ED. To avoid these limitations, further studies are necessary with larger samples and/or with randomized controlled methodology.

## Conclusion

Transdiagnostic DBT skills training groups seem to have positive outcomes at one year for individuals with BPD, BD and/or ADHD presenting with emotional dysregulation. These results are relevant in terms of DBT implementation, notably when evidence-based psychotherapies are difficult to implement due to access to services limitation. The transdiagnostic format can be more easily implemented outside of specialized settings for a specific disorder. Randomized controlled trials, observational studies on larger samples and cost-effectiveness studies are still needed.

## Data Availability

The results/data/figures in this manuscript have not been published elsewhere, nor are they under consideration (from you or one of your Contributing Authors) by another publisher.
